# Public awareness and healthcare professional advice for obesity as a risk factor for cancer in the UK: a cross-sectional survey

**DOI:** 10.1093/pubmed/fdx145

**Published:** 2017-11-14

**Authors:** Lucie Hooper, Annie S Anderson, Jack Birch, Alice S Forster, Gillian Rosenberg, Linda Bauld, Jyotsna Vohra

**Affiliations:** 1 Policy Research Centre for Cancer Prevention (PRCP), Cancer Research UK, Angel Building, 407 St. John Street, London, UK; 2 Centre for Research into Cancer Prevention and Screening, Level 7, Ninewells Hospital and Medical School, University of Dundee, Dundee, UK; 3 Department of Behavioural Science and Health, UCL, Gower Street, London, UK; 4 Cancer Research UK, Angel Building, 407 St. John Street, London, UK; 5 University of Stirling, Stirling, UK

**Keywords:** cancer, obesity, socio-economic factors

## Abstract

**Background:**

Overweight and obesity is the second biggest preventable cause of cancer after smoking, causing ~3.4 million deaths worldwide. This study provides current UK data on awareness of the link between obesity and cancer by socio-demographic factors, including BMI, and explores to what degree healthcare professionals provide weight management advice to patients.

**Methods:**

Cross-sectional survey of 3293 adults completed an online survey in February/March 2016, weighted to be representative of the UK population aged 18+.

**Results:**

Public awareness of the link between obesity and cancer is low (25.4% unprompted and 57.5% prompted). Higher levels of awareness existed for least deprived groups (*P* < 0.001), compared to more deprived groups. Most respondents had seen a healthcare practitioner in the past 12 months (91.6%) and 17.4% had received advice about their weight, although 48.4% of the sample were overweight/obese.

**Conclusion:**

Cancer is not at the forefront of people’s minds when considering health conditions associated with overweight or obesity. Socio-economic disparities exist in health knowledge across the UK population, with adults from more affluent groups being most aware. Healthcare professionals are uniquely positioned to provide advice about weight, but opportunities for intervention are currently under-utilized in healthcare settings.

## Background

Overweight and obesity is a contributor to diseases such as diabetes, coronary heart disease, stroke and cancer.^1^ In 2010 these conditions were estimated to have caused 3.4 million deaths and 4% of years of life lost worldwide.^2^ Latest data has found that 63% of the English^3^ and 67% of the Scottish adult population are overweight or obese.^4^

Obesity is associated with thirteen types of cancer,^5^ including breast (postmenopausal), kidney, bowel, and womb. In the UK 18 100 cancer cases are attributable to obesity, ~5% of all cancers.^6^ A recent study estimated that if current overweight and obesity trends continue in the UK there could be an additional 670 000 cancer cases by 2035.^7^

In the UK, few studies have explored public knowledge of the risks of obesity and associations with cancer. Reported levels of awareness have varied, perhaps due to differences in study design and recruitment approaches. One UK survey conducted in 2014 found that when asked an unprompted question only 10% of respondents recalled that being overweight is a risk factor for cancer.^8^ Latest WCRF data found 62% Britons to be aware of obesity as a risk factor for cancer.^9^

Research into cancer risk awareness amongst the UK population has shown differences in understanding between groups based on factors such as socio-economic status and ethnicity.^10–12^ However, there are few studies that examine how differences in body mass index (BMI) are related to awareness of overweight and obesity as a risk factor for cancer. In addition, few studies have examined cancer risk awareness and weight in the context of whether individuals have been given advice by healthcare professionals about the importance of maintaining a healthy weight. Previous research has suggested that overweight and obese adults are more likely to want to weigh less and attempt to lose weight after having received healthcare practitioner advice.^13^ Despite this, only a minority of patients report receiving advice on weight loss.^13^ Both patients and healthcare professionals have expressed frustrations with discussing advice regarding weight loss.^14^ Healthcare professionals may not be fully equipped^15,16^ to engage with patients about why weight is an important risk factor for a range of health conditions,^17^ including cancer.

Given these gaps in the literature this study aimed to investigate: public knowledge of health conditions linked to overweight and obesity, particularly cancer and the factors that influence this; and to determine whether adults in the UK who access healthcare services had been given advice about their weight.

## Methods

An online cross-sectional survey exploring knowledge of health risks associated with being overweight or obese was conducted in February 2016. A total of 3490 adults aged 18 and over were recruited by market research company, YouGov. The survey was weighted to be representative of the UK population. Geographically targeted over-samples of an additional 500 participants were obtained from Wales, Scotland and Northern Ireland to provide data relevant to these jurisdictions. There were 3293 complete responses to the survey (response rate = 94%). Participants were credited with 50 points (equivalent to 50p) to their YouGov account once the survey was completed.

### Measures

Questions included in the survey were informed by previous research conducted by Buykx *et al*.^18^ in Australia. Additional items were incorporated from other survey tools and adapted where necessary. Where no existing tools could be found, new questions were developed and tested for clarity, content and style of questions using Cancer Research UK’s patient panel group and health professionals from the Scottish Cancer Prevention Network.

Key demographic information held by YouGov included gender, age and region lived, including nine English regions, Wales, Scotland and Northern Ireland. Socio-economic status (SES) was calculated by YouGov based on the National Readership Survey (NRS) system and grouped into four: AB (higher and intermediate managerial, administrative, professional occupations), C1 (supervisory, clerical and junior managerial, administrative, professional occupations), C2 (skilled manual occupations), DE (semi-skilled and unskilled manual occupations, unemployed and lowest grade occupations). BMI was calculated using self-reported height and weight: weight (kg)/(height (m^2^)). BMI categories were grouped: underweight (<18.5 kg/m^2^), normal weight (18.5–24.9 kg/m^2^), overweight (25–29.9 kg/m^2^), obese (>30 kg/m^2^).^19^ Any cancer diagnosis was also recorded.

Cancer risk awareness was explored using an unprompted open-ended question: ‘Which, if any, health conditions do you think can result from being obese/ overweight?’; and a prompted question: ‘Which, if any, of the following health conditions do you think can result from being overweight/obese?’ Prompted response options included diabetes, heart disease, stroke, cancer and arthritis. Unprompted and prompted cancer awareness were both coded into dichotomous variables (aware of obesity as a risk factor for cancer versus not). Participants were provided with a list of thirteen cancer types and asked to select whether a person would be at increased risk of developing each cancer through being overweight or obese. ‘Yes’, ‘No’ and ‘Don’t know’ response options were provided. The four most prevalent cancer types in the UK associated with body weight were selected for analysis: breast (postmenopausal), kidney, bowel and womb. Responses were coded into two variables: ‘Yes’ if participants had selected the correct response, and ‘No’ if they incorrectly selected the cancer type. Don’t know responses were re-coded as ‘No’ responses.

Respondents were asked, ‘In the past 12 months, has a doctor, nurse, or other healthcare professional given you advice about your weight?’. Response options included: ‘No’, ‘Yes, lose weight’, ‘Yes, gain weight’, ‘Yes, maintain current weight’, ‘Not applicable, have not seen a healthcare professional in the past 12 months’, ‘Don’t know/can’t remember’. Advice to gain weight, other advice, not applicable and don’t know/can’t remember responses were coded as missing for the analysis.

### Analysis

Data were analysed using IBM SPSS Version 23 and Statacorp Stata Statistical Software release 13. Weights were applied to age, gender, SES and region to ensure the results were representative of the population. Weighted results are presented here, unless otherwise specified. Univariable chi squared analysis was undertaken to explore the relationship between cancer awareness and socio-demographic factors and BMI. Age, SES, gender, BMI and the cancer diagnosis variables were then entered into a multivariable logistic regression model, with step-wise elimination of non-significant variables. Ordinal regression was carried out to explore factors associated with receiving healthcare practitioner advice. As we are interested in advice given to overweight and obese people, the underweight BMI category was coded as missing for the regression analysis. Response categories: advice to gain weight; other advice; not applicable; and don’t know/can’t remember responses were coded as missing for the analysis.

## Results

A nationally representative sample of 3293 people from England, Wales, Scotland and Northern Ireland completed the survey. Of these, 51.3% were females and 48.7% were males. The proportion in each of the SES categories were: AB 22%; C1 30%; C2 15%, DE 33% (Table 1).

**Table 1:** Profile of sample population

	Unweighted sample (*n* = 3293)	Weighted sample (*n* = 3292)
	*n* (%)	*n* (%)
Gender		
Male	1580 (48)	1604 (48.7)
Female	1713 (52)	1689 (51.3)
Age		
18–39	1006 (30.5)	1202 (36.5)
40–59	1274 (38.7)	1126 (34.2)
60+	1013 (30.8)	965 (29.3)
Region of residence		
North East	89 (2.7)	135 (4.1)
North West	234 (7.1)	362 (11)
Yorkshire and the Humber	173 (5.3)	273 (8.3)
East Midlands	145 (4.4)	237 (7.2)
West Midlands	179 (5.4)	290 (8.8)
East of England	206 (6.3)	306 (9.3)
London	272 (8.3)	428 (13)
South East	294 (8.9)	451 (13.7)
South West	181 (5.5)	280 (8.5)
Wales	503 (15.3)	158 (4.8)
Scotland	513 (15.6)	280 (8.5)
Northern Ireland	504 (15.3)	92 (2.8)
Socio-economic status (SES)		
AB	913 (27.7)	724 (22)
C1	1037 (31.5)	988 (30)
C2	538 (16.3)	494 (15)
DE	805 (24.4)	1087 (33)
Body mass index (BMI)		
Underweight	75 (2.3)	85 (2.6)
Normal weight	1244 (37.8)	1327 (40.3)
Overweight	1015 (30.8)	944 (28.7)
Obese	700 (21.3)	648 (19.7)
Not calculated	259 (7.9)	290 (8.8)
Cancer diagnosis		
Ever been diagnosed with cancer	151 (4.8)	145 (4.6)
Never been diagnosed with cancer	3018 (95.2)	3009 (95.4)

Unprompted, only 25.4% of respondents listed cancer as a health condition that could result from being overweight or obese. When prompted with a list of potential health conditions, 57.5% selected cancer. Arthritis was the least selected health condition (50%) and diabetes was the most selected condition (93.6%).

For cancer types known to be associated with overweight and obesity, knowledge was wide-ranging with responses ranging from 21.5% for womb cancer to 60.1% for bowel cancer.

The vast majority of participants (3018/3293, 91.6%) had seen a seen a doctor, nurse or healthcare professional in a healthcare setting in the past 12 months and recalled whether or not they had received advice about their weight. Of these, 74.2% did not receive any advice and 17.4% had received some form of advice about their weight. Twelve percent had been told to lose weight; 4% to maintain their current weight; 1% to gain weight and the remaining 1% were given ‘other advice’. As the proportion of the sample given advice to gain weight or other advice was so small, they were excluded for the next stage of the analysis. The majority of the unweighted sample (52.1%) and just under half of the weighted sample (48.4%) were overweight or obese. Results examining the relationship between the weight of respondent and receipt of advice are outlined below.

### Univariate analysis

For the unprompted cancer awareness question, it was found that SES (30.1% AB versus 22% DE *P* < 0.001) and having ever had a cancer diagnosis were significant factors in cancer awareness (34.5% ever diagnosed versus 24.7% never diagnosed *P* = 0.008). Gender also had a small but significant association with awareness (26.9% females versus 23.8% males *P* = 0.041).

Prompted awareness was significantly associated with SES and BMI. AB respondents were more likely to be aware of being overweight or obese as a risk factor for cancer than all of the other social grades (66.6 versus 50.6% *P* < 0.001). Respondents of normal weight were the most likely to be aware of overweight/obesity as a risk factor for cancer and respondents who were obese were the least likely to be aware (63.6 versus 52.3%, *P* < 0.001).

Of the four cancer types examined, awareness of their association with overweight/obesity was greatest from the highest SES group (AB) across all cancer types (*P* < 0.001). People who were normal weight were most likely to know of the relationship between all cancer types and obesity (*P* < 0.001) apart from womb cancer (*P* = 0.065) Highest awareness was found among 18–39 year olds for kidney and womb cancers (*P* < 0.001) and there was greater awareness among females for postmenopausal breast cancer (34 versus 28.1%, *P* < 0.001) and males for kidney cancer (46.2 versus 42%, *P* = 0.008).

All demographics listed: age, gender, SES, including BMI and cancer diagnosis were significant factors in receiving advice to lose or maintain weight. Around twice as many people aged over 60 received advice to lose weight compared to 18–39 year olds (16.1 versus 7.8% *P* < 0.001). Obese and overweight respondents were significantly more likely to receive advice to lose weight than those who were normal weight (obese: 38.3% and overweight: 11.7% versus normal weight 2.3% *P* < 0.001).

### Multivariate analysis

The logistic regression models for unprompted cancer awareness showed that the highest SES group (AB) were more likely to be aware of the links between overweight and cancer than those from the lowest two SES groups (C2 *P* < 0.001, OR = 0.532 and DE *P* < 0.001, OR = 0.638) as were males (compared to females) (*P* = 0.012, OR = 1.233). Having ever been diagnosed with cancer was also shown to be significant contributor to unprompted cancer awareness of obesity risk (*P* = 0.017, OR = 0.648) (Table 2).

**Table 2:** Multivariate logistic regression results for unprompted and prompted awareness of the link between cancer and overweight/obesity

	Awareness of cancer (unprompted)				Awareness of cancer (prompted)			
	Yes (%)	OR	CI (95%)	*P*-value	Yes (%)	OR	CI (95%)	*P*-value
Overall (*n* = 3293)	25.4	–	–	–	57.5	–	–	–
Gender								
Male (*n* = 1604)	23.8	–	–	–	58	–	–	–
Female (*n* = 1690)	**26.9**	**1.233**	**1.047–1.451**	**0.012**	57	0.985	0.854–1.136	0.839
Age								
18–39 (*n* = 1202)	26.4	–	–	–	59.4	–	–	–
40–59 (*n* = 1126)	25	0.983	0.809–1.195	0.866	56.4	0.945	0.796—1.122	0.519
60+ (*n* = 965)	24.8	0.951	0.772–1.171	0.636	56.4	0.953	0.794–1.143	0.603
Social grade								
AB (*n* = 724)	30.1	–	–	–	66.6	–	–	–
C1 (*n* = 988)	28.8	0.927	0.746–1.151	0.491	**60.5**	**0.77**	**0.631–0.941**	**0.011**
C2 (*n* = 494)	**19.2**	**0.532**	**0.402–0.705**	**<0.001**	**53.2**	**0.573**	**0.453–0.725**	**<0.001**
DE (*n* = 1087)	**22**	**0.638**	**0.512–0.795**	**<0.001**	**50.6**	**0.515**	**0.424–0.626**	**<0.001**
BMI								
Underweight (*n* = 85)	26.2	–	–	–	58.3	–	–	–
Normal Weight (*n* = 1327)	27.9	1.078	0.647–1.794	0.773	63.6	1.165	0.731–1.856	0.522
Overweight (*n* = 944)	25.5	0.977	0.581–1.642	0.931	58.1	0.937	0.583–1.503	0.786
Obese (648)	23.5	0.835	0.490–1.423	0.508	52.3	0.764	0.472–1.237	0.273
Diagnosed with cancer								
Ever been diagnosed with cancer (*n* = 137)	34.5	–	–	–	60.7	–	–	–
Never been diagnosed with cancer (*n* = 2684)	**24.7**	**0.648**	**0.454–0.924**	**0.017**	56.8	0.88	0.624–1.241	0.467

Values highlighted in bold are *P* < 0.05.

For prompted cancer awareness, the only factor independently associated with awareness was SES. Those from the highest social grade (AB) showed greatest awareness compared to all other SES groups (C1 *P* = 0.11, OR = 0.77, C2 *P* < 0.001, OR = 0.573, DE *P* < 0.001, OR = 0.515) (Table 3).

**Table 3:** Multivariate logistic regression results for awareness of the association between overweight/obesity and four cancer types

Cancer type awareness																
	Postmenopausal breast				Kidney				Bowel				Womb			
	Yes (%)	OR	CI	*P*-value	Yes (%)	OR	CI	*P*-value	Yes (%)	OR	CI	*P*-value	Yes (%)	OR	CI	*P*-value
Gender																
Male (*n* = 1604)	28.1	–	–	–	46.6	–	–	–	60.3	–	–	–	21.2	–	–	–
Female (*n* = 1690)	**34.0**	**0.746**	**0.638–0.873**	**<0.001**	**42.0**	**1.177**	**1.024–1.354**	**0.022**	59.9	0.983	0.844–1.146	0.828	21.8	0.995	0.832–1.187	0.956
Age																
18–39 (*n* = 1202)	32.9	–	–	–	53.1	–	–	–	62.6	–	–	–	25.5	–	–	–
40–59 (*n* = 1126)	30.3	1.047	0.862–1.271	0.642	**39.6**	**1.722**	**1.458–2.035**	**<0.001**	58.1	1.077	0.894–1.297	0.437	**20.7**	**1.384**	**1.123–1.705**	**0.002**
60+ (*n* = 965)	29.9	1.108	0.902–1.361	0.329	**38.8**	**1.759**	**1.476**–**2.096**	**<0.001**	59.3	0.962	0.790–1.173	0.704	**17.5**	**1.757**	**1.400**–**2.205**	**<0.001**
Social grade																
AB (*n* = 724)	36.5	–	–	–	49.7	–	–	–	68.0	–	–	–	24.6	–	–	–
C1 (*n* = 988)	32.9	1.108	0.898–1.367	0.340	**46.5**	**1.259**	**1.035**–**1.531**	**0.021**	61.5	1.227	0.992–1.517	0.059	**19.9**	**1.291**	**1.012–1.646**	**0.040**
C2 (*n* = 494)	**28.1**	**1.436**	**1.109–1.861**	**0.006**	47.0	1.109	0.880–1.398	0.380	62.1	1.178	0.915–1.515	0.203	**17.6**	**1.469**	**1.088**–**1.984**	**0.012**
DE (*n* = 1087)	**27.3**	**1.506**	**1.217**–**1.863**	**<0.001**	**37.4**	**1.634**	**1.348**–**1.981**	**<0.001**	**51.5**	**1.836**	**1.494**–**2.257**	**<0.001**	22.8	1.044	0.826–1.319	0.719
BMI																
Underweight (*n* = 85)	23.5	–	–	–	47.1	–	–	–	**48.2**	–	–	–	12.9	–	–	–
Normal Weight (*n* = 1327)	**33.8**	**0.557**	**0.331**–**0.937**	**0.028**	48.6	0.882	0.563–1.381	0.582	**64.4**	**0.510**	**0.327**–**0.794**	**0.003**	**23.4**	**0.448**	**0.232**–**0.863**	**0.016**
Overweight (*n* = 944)	**33.4**	**0.549**	**0.324**–**0.931**	**0.026**	47.7	0.849	0.538–1.341	0.484	**62.3**	**0.563**	**0.359**–**0.882**	**0.012**	**21.5**	**0.461**	**0.237**–**0.897**	**0.023**
Obese (648)	25.0	0.822	0.480–1.410	0.477	36.9	1.263	0.792–2.014	0.326	56.2	0.694	0.439–1.097	0.118	19.9	0.510	0.260–1.002	0.051
Diagnosed with cancer																
Ever been diagnosed with cancer (*n* = 137)	36.6	–	–	–	40.0	–	–	–	55.9	–	–	–	19.3	–	–	–
Never been diagnosed with cancer (*n* = 2684)	31.0	1.271	0.881–1.833	0.199	44.1	0.966	0.671–1.390	0.852	59.6	0.843	0.590–1.205	0.349	21.5	1.063	0.677–1.668	0.791

Values highlighted in bold are *P* < 0.05.

Gender was an independent predictor for cancer type awareness for postmenopausal breast and kidney cancer with females (*P* < 0.001, OR = 0.746) and males (*P* = 0.022, OR = 1.177) more likely to be aware of body weight association, respectively. Those from the highest SES group (AB) were most likely to be aware of each cancer type, compared to all other groups (postmenopausal breast: C2 *P* = 0.006 OR = 1.436, DE *P* < 0.001, OR = 1.506; kidney: C1 *P* = 0.021, OR = 1.259, DE *P* < 0.001, OR = 1.634; bowel: DE *P* < 0.001 OR = 1.836; womb: C1 *P* = 0.040. OR = 1.291, C2 *P* = 0.012, OR = 1.469). Those aged 18–39 years old were most likely to know that kidney (40–59 *P* < 0.001, OR = 1.722, 60 + *P* < 0.001 OR = 1.759) and womb (40–59 *P* = 0.002, OR = 1.384, 60 + *P* < 0.001, OR = 1.757) cancers are associated with overweight and obesity compared to all other age groups (Table 3).

Ordinal regression analysis for healthcare practitioner advice found all factors were significant apart from age and having previously received a cancer diagnosis. Normal weight respondents were significantly less likely to receive advice to lose weight, but were more likely to be told to maintain weight than overweight or obese respondents (*P* < 0.001 OR = 5.844) (Table 4).

**Table 4:** Ordinal logistic regression results for receiving healthcare practitioner advice regarding weight

	No %	Yes, lose weight %	Yes, maintain weight %	OR	CI (95%)	*P* value
Overall (*n* = 2953)	82.8	13.2	4.1	–	–	–
Gender (*n* = 2953)						
Male	**80.8**	**14.6**	**4.6**	**0.735**	**0.591–0.914**	**0.006**
Female	84.6	11.8	3.5	–	–	–
Age (*n* = 2593)						
18–39	89.2	7.8	3.1	1.904	1.416–2.558	0.939
40–59	79.5	16.5	3.9	1.139	0.886–1.464	0.381
60+	78.5	16.1	5.4	–	–	–
SES (*n* = 2593)						
AB	**81.8**	**13.4**	**4.7**	**0.645**	**0.477–0.873**	**0.005**
C1	**82.5**	**12.3**	**5.3**	**0.576**	**0.432–0.768**	<**0.001**
C2	**80.1**	**15.8**	**4.1**	**0.63**	**0.452–0.877**	**0.006**
DE	84.8	12.7	2.5	–	–	–
BMI (*n* = 2646)						
Normal weight	**92**	**2.3**	**5.7**	**5.844**	**4.435–7.701**	<**0.001**
Overweight	**83.4**	**11.7**	**4.9**	**3.158**	**2.444–4.082**	<**0.001**
Obese	61.2	38.3	0.5	–	–	–
Cancer diagnosis (*n* = 2821)						
Yes	77.4	13.1	9.5	0.784	0.497–1.236	0.295
No	82.9	13.3	4	–	–	–

Values highlighted in bold are P < 0.05.

## Discussion

### Main finding of this study

Awareness of being overweight or obese as a risk factor for cancer was generally low with only 25.4% of respondents listing cancer when asked an unprompted question. This shows that cancer is not at the forefront of people’s minds when considering health risks associated with body weight. When asked a prompted question, 57.5% of the sample recognized overweight/obesity as a risk factor for cancer; however, awareness of the association between overweight and diabetes was much greater (93.6%). In both instances, SES played a significant role with those from the highest SES group being more aware than the other SES groups.

There were misconceptions about cancer types linked to overweight and obesity. Greater levels of awareness were found for cancers of the digestive system organs such as, bowel and kidney, but not for reproductive organs, such as womb or postmenopausal breast.

The majority of respondents (91.6%) had seen a healthcare practitioner in the past 12 months. However, only one in five respondents had been given any advice about their weight and if advice was provided, it was focused on losing weight. It is positive that the group receiving advice to lose weight most often self-reported as being obese (38.3%) but this was not the case individuals who were overweight (11.7%). It is important that advice on weight is provided by health professionals to individuals who are overweight and therefore at higher risk of becoming obese. It is estimated that average weight gain in adulthood is around 400 g per annum and this may be greater with increasing age.^20^

### What is already known about this topic

Although previous studies have collected data on awareness of obesity as a risk factor for cancer,^8,9,11,12,21^ there are limited data available for unprompted responses and particularly data exploring knowledge of risks related to specific cancer types. Public awareness of disease risk is considered important as it has an influence on behavioural intentions.^22^

A recent study reported that 17% of overweight and 42% of obese people had received advice to lose weight from a healthcare practitioner.^13^ A 2013 literature review and meta-analysis of survey data found physician advice had a significant impact on intention to change behaviour and lose weight.^23^ Healthcare settings are favourable environments for receiving weight loss advice^13,24^ and effecting behaviour change^23,25,26^ as well as raising cancer risk awareness.^22^ However, such opportunities are currently under-utilized.^27^

### What the study adds

This is the first study to explore factors associated with knowledge of obesity as a risk factor for cancer using both prompted and unprompted response options. Additionally, data on public awareness of the link between overweight/obesity and cancer for the four most prevalent cancer types linked to overweight and obesity has been explored. Those from lower SES groups are more likely to be overweight or obese (64% males and 51% females in highest SES group compared to 67% males and 63% females in lowest SES group).^28^ Modelled projections show overweight and obesity trends will increase by 2035 (76% males and 69% females) and the gap between the highest and lowest income quintile is expected to widen further.^7^ This study lends support to the concept of providing targeted programmes for vulnerable populations. Whilst there is concern over health education increasing health inequalities this has not been demonstrated in weight loss programmes.^29^

The data reported here show that there are public misconceptions of cancer types associated with overweight and obesity. These are of particular interest as in the UK breast cancer is one of the most prevalent cancers,^30^ yet there are poor levels of public awareness of its link to body weight. In addition, there has also been an increase in cancers of the womb that has been linked to increases in population obesity rates.^31^ The proportion of kidney cancer cases are also increasing and are projected to continue.^30^ It is therefore vital that population measures to halt and ultimately reduce rates for overweight and obese are implemented in the UK.

This study reiterates similar data from earlier research^13,27^ exploring healthcare practitioner advice about body weight, therefore building the evidence base further in this area. The data has shown that whilst most of the population see a health practitioner, a small proportion are given any advice about their weight. As there is a real need to prevent people from becoming overweight or obese, attendance at healthcare settings provide significant opportunities to advise people about their weight.^13,23,25,26^ However, these are currently being missed.^27^ There is a need for healthcare practitioners to be sufficiently equipped and trained to provide weight loss advice.^15,16,32^

### Limitations of this study

Limitations include the sampling method which was an online survey where a panel was used to recruit participants and therefore a self-selected group. As with any cross-sectional survey, data cannot be collected over a period time to analyse behaviour change. When comparing the study data to Health Survey for England (HSE) data, the levels of obesity, as calculated by self-reported BMI, are lower in this study than those seen in the latest HSE (2015) data (20 versus 27%).^28^ This could be due to 9% of participants not providing their weight and that the data was self-reported. HSE use an objective method of measuring weight and height at the time of collecting survey data and is therefore a more validated approach. A priority for future research should be to explore what kind of advice healthcare practitioners are giving to patients about their weight, whether these are in line with official guidance and what the barriers are to giving advice.

### Summary

Only a quarter of adults in the UK are aware of cancer as a health condition associated with being overweight or obese. Differences in awareness exist between socio-economic groups and weight status, as those from more affluent groups have higher levels of awareness and generally lower BMI than those from the least affluent groups. Opportunities exist for healthcare professionals to advise about weight loss, but these appear to be under-utilized—in this study only 38.3% of respondents who were obese were advised to lose weight.

## Supplementary Material

Supplementary Data
