# Supplementary material for: Public awareness and healthcare professional advice for obesity as a risk factor for cancer in the UK: a cross-sectional survey
Source: J Public Health (Oxf). 2017 Nov 14;40(4):797–805. doi: 10.1093/pubmed/fdx145 (PMC6306085; doi:10.1093/pubmed/fdx145)
Supplement: Supplementary Data [file supplementary_material1_survey.pdf]

# Questionnaire UK1041765_CRUK_Risk

Valued Customer

Version unknown

2016-02-29 22:40:02.737000+00:00

Page: implicit_page_q15 .... 1
Page: implicit_page_q1 .... 2
Page: implicit_page_q2 .... 3
Page: implicit_page_q3 .... 4
Page: implicit_page_q4 .... 5
Module: time if q4_1 in [2,3,4,5,6,7,8] or q4_2 in [2,3,4,5,6,7,8] or q4_3 in [2,3,4,5,6,7,8] or q4_3 in [2,3,4,5,6,7,8] .... 5
Page: walking if q4_1 in [2,3,4,5,6,7,8] .... 5
Page: moderate if q4_2 in [2,3,4,5,6,7,8] .... 9
Page: vigorous if q4_3 in [2,3,4,5,6,7,8] .... 13
Page: strength if q4_4 in [2,3,4,5,6,7,8] .... 17
Module: implicit_module_1 .... 21
Page: implicit_page_q6 .... 22
Page: implicit_page_q7a .... 23
Page: calories if q7a==1 .... 23
Page: implicit_page_q8a .... 24
Page: freesugar if q8a==1 .... 24
Page: implicit_page_q8e .... 25
Page: implicit_page_q9a .... 25
Page: child_freesugar if q9a==1 .... 25
Page: implicit_page_q10 .... 26
Module: sugar .... 26
Page: cola .... 27
Page: ice .... 27
Page: choco .... 27
Page: latte .... 27
Page: ginger .... 28
Page: energy .... 28
Module: implicit_module_2 .... 28
Page: implicit_page_q12 .... 29
Page: implicit_page_q13 .... 29
Page: implicit_page_q14 .... 30
Page: implicit_page_q16a .... 31
Page: implicit_page_height_metres .... 37
Page: implicit_page_q18 .... 39

Page: implicit_page_q19 ..... 39
Page: implicit_page_q20 ..... 40
Page: implicit_page_q21 ..... 40
Page: alcohol if q21!=1 ..... 40
Page: implicit_page_q23 ..... 41
Page: end ..... 41

# Page: implicit_page_q15

**q15- *required*** GRID

The following may or may not increase a person’s chance of developing cancer. We are interested in your opinion. For each of the following, please say whether or not you think it could increase a person’s chance of developing cancer.

| | |
|---|---|
| required | HARD |
| displaymax | 6 |
| roworder | randomize |

ROWS

| | |
|---|---|
| q15_1- *required* | Smoking |
| q15_2- *required* | Being overweight |
| q15_3- *required* | Having a close relative with cancer |
| q15_4- *required* | Drinking alcohol |
| q15_5- *required* | NOT doing much physical activity |
| q15_6- *required* | Getting sunburnt |
| q15_7- *required* | Eating too much red meat |
| q15_8- *required* | Exposure to another person's smoking |
| q15_9- *required* | Being older |
| q15_10- *required* | Being infected with HPV (human papillomavirus) |
| q15_11- *required* | Not breastfeeding (for mothers) |
| q15_12- *required* | Gaining weight in adult life |
| q15_13- *required* | Eating too much processed meat |
| q15_14- *required* | Not eating enough fibre |
| q15_15- *required* | Not eating many fruits or vegetables |
| q15_16- *required* | Not taking vitamin supplements |
| q15_17- *required* | Living by electromagnetic fields |

COLUMNS

1 ○ Yes it could
2 ○ No it couldn't
99 ○ Don't know / not sure
8 *Skipped*
9 *Not Asked*

## Page: implicit_page_q1

***q1- required*** GRID

How often do you have food....

| | |
|---|---|
| required | HARD |
| transpose | True |

ROWS

| | |
|---|---|
| q1_1- *required* | At home such as ready meals, burgers, pizza, or chips? |
| q1_2- *required* | From takeaway places like McDonalds, Burger King, Pizza Hut, KFC, or local takeaway food places? |

COLUMNS

1 ○ 2 to 3 times a day
2 ○ Once a day
3 ○ 5 or 6 times a week
4 ○ 2 to 4 times a week
5 ○ Once a week
6 ○ 1 to 3 times per month
7 ○ Less than once a month
8 ○ Never
8 *Skipped*
9 *Not Asked*

# Page: implicit_page_q2

**q2-*required***

How often do you …

GRID

| | |
|---|---|
| required | HARD |
| transpose | True |

ROWS

| | |
|---|---|
| q2_1- *required* | Drink soft drinks such as cola, cordials, sports drinks or energy drinks (do not include sugar free drinks)? |
| q2_2- *required* | Eat confectionery (such as sweets and chocolates), cakes, muffins, sweet pies, pastries or biscuits? |

COLUMNS

1 ○ 6 or more times a day
2 ○ 4 or 5 times a day
3 ○ 2 to 3 times a day
4 ○ Once a day
5 ○ 5 or 6 times a week
6 ○ 2 to 4 times a week
7 ○ Once a week
8 ○ 1 to 3 times per month
9 ○ Less than once a month
10 ○ Never
8 *Skipped*
9 *Not Asked*

## Page: implicit_page_q3

**q3-** ***required*** GRID

To what extent do you agree the following have responsibility for tackling the increasing rates of obesity?

required HARD

roworder randomize

ROWS

| | |
|---|---|
| q3_1- *required* | National Government |
| q3_2- *required* | Local Government |
| q3_3- *required* | Charities |
| q3_4- *required* | Schools |
| q3_5- *required* | The NHS |
| q3_6- *required* | Individuals |
| q3_7- *required* | Parents |
| q3_8- *required* | The food industry (e.g. manufacturers, suppliers, retailers) |
| q3_9- *required* | The drinks industry (e.g. manufacturers, suppliers, retailers) |

COLUMNS

1 ○ Strongly disagree
2 ○ Disagree
3 ○ Neither agree nor disagree
4 ○ Agree
5 ○ Strongly agree
8 *Skipped*
9 *Not Asked*

## Page: implicit_page_q4

***q4- required*** GRID

In the past week on how many _days_, if any, did you do the following activities for at least 10 minutes at a time?

required HARD

ROWS

| | |
|---|---|
| q4_1- *required* | Walking, this includes at work and at home, walking to travel from place to place |
| q4_2- *required* | Moderate physical activity such as cycling or brisk walking |
| q4_3- *required* | Vigorous physical activity, such as running, singles tennis, running, swimming or football |
| q4_4- *required* | Strength exercises that work all the major muscles such as dancing, yoga, lifting weights |

COLUMNS

1 ○ 0
2 ○ 1
3 ○ 2
4 ○ 3
5 ○ 4
6 ○ 5
7 ○ 6
8 ○ 7
9 ○ Not sure
8 *Skipped*
9 *Not Asked*

end module: implicit_module_0

# Module: time if q4_1 in [2,3,4,5,6,7,8] or q4_2 in [2,3,4,5,6,7,8] or q4_3 in [2,3,4,5,6,7,8] or q4_3 in [2,3,4,5,6,7,8]

## Page: walking if q4_1 in [2,3,4,5,6,7,8]

You ticked that you had done the following for at least 10 minutes on at least one day in the last week... <b>walking, this includes at work and at home, walking to travel from place to place</b> How much _time_ do you normally spend doing this activity on _one of those days_?

**q5_1a_dd-*required***

DROPDOWN

Hours

required HARD

1 ○ 0
2 ○ 1
3 ○ 2
4 ○ 3
5 ○ 4
6 ○ 5
7 ○ 6
8 ○ 7
9 ○ 8
10 ○ 9
11 ○ 10
12 ○ 11
13 ○ 12
14 ○ 13
15 ○ 14
16 ○ 15
17 ○ 16
18 ○ 17
19 ○ 18
20 ○ 19
21 ○ 20
22 ○ 21
23 ○ 22
24 ○ 23
25 ○ 24
98 *Skipped*
99 *Not Asked*

| **q5_1b_dd-*required*** Minutes | DROPDOWN |
|---|---|

| | |
|---|---|
| required | HARD |

○ 0
○ 1
○ 2
○ 3
○ 4
○ 5
○ 6
○ 7
○ 8
○ 9
○ 10
○ 11
○ 12
○ 13
○ 14
○ 15
○ 16
○ 17
○ 18
○ 19
○ 20
○ 21
○ 22
○ 23
○ 24
○ 25
○ 26
○ 27
○ 28
○ 29
○ 30
○ 31
○ 32
○ 33
○ 34
○ 35
○ 36
○ 37
○ 38
○ 39
○ 40
○ 41
○ 42
○ 43
○ 44
○ 45
○ 46
○ 47

49 ○ 48
50 ○ 49
51 ○ 50
52 ○ 51
53 ○ 52
54 ○ 53
55 ○ 54
56 ○ 55
57 ○ 56
58 ○ 57
59 ○ 58
60 ○ 59
61 ○ 60
98 *Skipped*
99 *Not Asked*

# Page: moderate if q4_2 in [2,3,4,5,6,7,8]

You ticked that you had done the following for at least 10 minutes on at least one day in the last week ... <b>moderate aerobic activity such as cycling or fast walking</b> How much _time_ do you normally spend doing this activity on _one of those days_?

**q5_2a_dd-*required***

DROPDOWN

Hours

required HARD

1 ○ 0
2 ○ 1
3 ○ 2
4 ○ 3
5 ○ 4
6 ○ 5
7 ○ 6
8 ○ 7
9 ○ 8
10 ○ 9
11 ○ 10
12 ○ 11
13 ○ 12
14 ○ 13
15 ○ 14
16 ○ 15
17 ○ 16
18 ○ 17
19 ○ 18
20 ○ 19
21 ○ 20
22 ○ 21
23 ○ 22
24 ○ 23
25 ○ 24
98 *Skipped*
99 *Not Asked*

**q5_2b_dd-*required*** DROPDOWN

Minutes

| | |
|---|---|
| required | HARD |

1 ○ 0
2 ○ 1
3 ○ 2
4 ○ 3
5 ○ 4
6 ○ 5
7 ○ 6
8 ○ 7
9 ○ 8
10 ○ 9
11 ○ 10
12 ○ 11
13 ○ 12
14 ○ 13
15 ○ 14
16 ○ 15
17 ○ 16
18 ○ 17
19 ○ 18
20 ○ 19
21 ○ 20
22 ○ 21
23 ○ 22
24 ○ 23
25 ○ 24
26 ○ 25
27 ○ 26
28 ○ 27
29 ○ 28
30 ○ 29
31 ○ 30
32 ○ 31
33 ○ 32
34 ○ 33
35 ○ 34
36 ○ 35
37 ○ 36
38 ○ 37
39 ○ 38
40 ○ 39
41 ○ 40
42 ○ 41
43 ○ 42
44 ○ 43
45 ○ 44
46 ○ 45
47 ○ 46
48 ○ 47

49 ○ 48
50 ○ 49
51 ○ 50
52 ○ 51
53 ○ 52
54 ○ 53
55 ○ 54
56 ○ 55
57 ○ 56
58 ○ 57
59 ○ 58
60 ○ 59
61 ○ 60
98 *Skipped*
99 *Not Asked*

# Page: vigorous if q4_3 in [2,3,4,5,6,7,8]

You ticked that you had done the following for at least 10 minutes on at least one day in the last week ... <b>vigorous aerobic activity, such as running, singles tennis, running, swimming or football</b> How much _time_ do you normally spend doing this activity on _one of those days_?

**q5_3a_dd-*required***

Hours

DROPDOWN

required HARD

1 ○ 0
2 ○ 1
3 ○ 2
4 ○ 3
5 ○ 4
6 ○ 5
7 ○ 6
8 ○ 7
9 ○ 8
10 ○ 9
11 ○ 10
12 ○ 11
13 ○ 12
14 ○ 13
15 ○ 14
16 ○ 15
17 ○ 16
18 ○ 17
19 ○ 18
20 ○ 19
21 ○ 20
22 ○ 21
23 ○ 22
24 ○ 23
25 ○ 24
98 *Skipped*
99 *Not Asked*

**q5_3b_dd-*required*** DROPDOWN

Minutes

| | |
|---|---|
| required | HARD |

- ○ 0
- ○ 1
- ○ 2
- ○ 3
- ○ 4
- ○ 5
- ○ 6
- ○ 7
- ○ 8
- ○ 9
- ○ 10
- ○ 11
- ○ 12
- ○ 13
- ○ 14
- ○ 15
- ○ 16
- ○ 17
- ○ 18
- ○ 19
- ○ 20
- ○ 21
- ○ 22
- ○ 23
- ○ 24
- ○ 25
- ○ 26
- ○ 27
- ○ 28
- ○ 29
- ○ 30
- ○ 31
- ○ 32
- ○ 33
- ○ 34
- ○ 35
- ○ 36
- ○ 37
- ○ 38
- ○ 39
- ○ 40
- ○ 41
- ○ 42
- ○ 43
- ○ 44
- ○ 45
- ○ 46
- ○ 47

49 ○ 48
50 ○ 49
51 ○ 50
52 ○ 51
53 ○ 52
54 ○ 53
55 ○ 54
56 ○ 55
57 ○ 56
58 ○ 57
59 ○ 58
60 ○ 59
61 ○ 60
98 *Skipped*
99 *Not Asked*

## Page: strength if q4_4 in [2,3,4,5,6,7,8]

You ticked that you had done the following for at least 10 minutes on at least one day in the last week ... <b>strength exercises that work all the major muscles (legs, hips, back, abdomen, chest, shoulders and arms), such as dancing, yoga, lifting weights</b> How much _time_ do you normally spend doing this activity on _one of those days_?

**q5_4a_dd-*required*** DROPDOWN

Hours

required HARD

1 ○ 0
2 ○ 1
3 ○ 2
4 ○ 3
5 ○ 4
6 ○ 5
7 ○ 6
8 ○ 7
9 ○ 8
10 ○ 9
11 ○ 10
12 ○ 11
13 ○ 12
14 ○ 13
15 ○ 14
16 ○ 15
17 ○ 16
18 ○ 17
19 ○ 18
20 ○ 19
21 ○ 20
22 ○ 21
23 ○ 22
24 ○ 23
25 ○ 24
98 *Skipped*
99 *Not Asked*

| **q5_4b_dd-*required*** | DROPDOWN |
| --- | --- |
| Minutes | |

| | |
| --- | --- |
| required | HARD |

○ 0
○ 1
○ 2
○ 3
○ 4
○ 5
○ 6
○ 7
○ 8
○ 9
○ 10
○ 11
○ 12
○ 13
○ 14
○ 15
○ 16
○ 17
○ 18
○ 19
○ 20
○ 21
○ 22
○ 23
○ 24
○ 25
○ 26
○ 27
○ 28
○ 29
○ 30
○ 31
○ 32
○ 33
○ 34
○ 35
○ 36
○ 37
○ 38
○ 39
○ 40
○ 41
○ 42
○ 43
○ 44
○ 45
○ 46
○ 47

49 ○ 48
50 ○ 49
51 ○ 50
52 ○ 51
53 ○ 52
54 ○ 53
55 ○ 54
56 ○ 55
57 ○ 56
58 ○ 57
59 ○ 58
60 ○ 59
61 ○ 60
98 *Skipped*
99 *Not Asked*

end module: time if q4_1 in [2,3,4,5,6,7,8] or q4_2 in [2,3,4,5,6,7,8] or q4_3 in [2,3,4,5,6,7,8] or q4_3 in [2,3,4,5,6,7,8]

# Module: implicit_module_1

# Page: implicit_page_q6

**q6- *required*** DYNAMIC GRID

To reduce obesity rates in the UK, to what extent would you support or oppose the following?

| | |
|---|---|
| required | HARD |
| roworder | randomize |

ROWS

| | |
|---|---|
| q6_1- *required* | The NHS providing weight management programmes |
| q6_2- *required* | The government introducing a tax on sugary drinks |
| q6_3- *required* | The food industry reducing average portion sizes of high calorie foods |
| q6_4- *required* | The government restricting advertising of high calorie food on TV |
| q6_5- *required* | The government providing free school meals to all students under 18 years |
| q6_6- *required* | Local government making towns and cities more cycle-friendly |
| q6_7- *required* | Local government making towns and cities more accessible to encourage walking |
| q6_8- *required* | Retailers/producers restricting promotions (e.g. two for one) on high calorie food and drinks |
| q6_9- *required* | The government launching a public health campaign to promote healthy eating and physical activity |
| q6_10- *required* | The government introducing a tax on high fat content foods |
| q6_11- *required* | Schools increasing the amount of physical activity in the curriculum |
| q6_12- *required* | Supermarkets allocating healthier products at the end of aisles and checkouts |
| q6_13- *required* | The government increasing access to community sports facilities and programmes |
| q6_14- *required* | The NHS providing free access to take part in sports |
| q6_15- *required* | Restaurants labelling menus with nutritional and calorie information |
| q6_16- *required* | Local government introducing restrictions on food takeaways near schools |
| q6_17- *required* | Manufacturers reducing sugar content in food and drinks |
| q6_18- *required* | Manufacturers reducing fat content in food and drinks |

COLUMNS

1 ○ Strongly oppose
2 ○ Oppose
3 ○ Neither support or oppose
4 ○ Support
5 ○ Strongly support
6 ○ Don't know
8 *Skipped*
9 *Not Asked*

## Page: implicit_page_q7a

**q7a-*required*** SINGLE CHOICE

Do you know how many calories it is recommended someone like you should consume each day?

required HARD

1 ○ Yes
2 ○ No
3 ○ I'm not sure what calories are
8 *Skipped*
9 *Not Asked*

## Page: calories if q7a==1

**q7b_1-*required*** OPEN REAL TEXTBOX

Please type in how many calories you think are recommended each day for a person like you.

required HARD

cols 5

**q7c-*required*** SINGLE CHOICE

Do you use this number of calories to keep track of how many you consume on a daily basis?

required HARD

1 ○ Every day
2 ○ Sometimes
3 ○ Rarely
4 ○ Never
8 *Skipped*
9 *Not Asked*

## Page: implicit_page_q8a

**q8a-*required*** SINGLE CHOICE

Do you know how much added or free sugar it is recommended you should not exceed in your daily dietary energy intake?  Free sugars are any sugars added to food or drinks, or found naturally in honey, syrups and unsweetened fruit juices.

| | |
|---|---|
| required | HARD |

1 ○ Yes
2 ○ No
3 ○ Don't know
8 *Skipped*
9 *Not Asked*

## Page: freesugar if q8a==1

<div class="alert alert-error">Please only select one of these options</div>

<div class="alert alert-error">Please select one of these options</div>

Please type in how much added or free sugars it is recommended you should not exceed as part of your daily dietary energy intake.</br><br/>Please use just one of the boxes below . <br><br>

**q8b_1** OPEN REAL TEXTBOX

Please type in the percentage (%).

| | |
|---|---|
| required | NONE |
| max | 100 |
| min | 0 |
| right | % |

<b> Or</b>

**q8c_1** OPEN INTEGER TEXTBOX

Please type in the number of grams of sugar per day.

| | |
|---|---|
| required | NONE |
| min | 0 |
| max | 10000 |
| right | g |

<b> Or</b>

**q8d_1** OPEN REAL TEXTBOX

Please type in the number of teaspoons of sugar per day.

| | |
|---|---|
| required | NONE |
| max | 1000 |
| min | 0 |
| right | tsp |

# Page: implicit_page_q8e

**q8e- *Show if q8a==1/required*** SINGLE CHOICE

Do you use this $q8_added_free_sugar_text to track how much sugar you consume on a daily basis?

required HARD

1 ○ Every day
2 ○ Sometimes
3 ○ Rarely
4 ○ Never
8 *Skipped*
9 *Not Asked*

# Page: implicit_page_q9a

**q9a- *required*** SINGLE CHOICE

Do you know how much added or free sugar it is recommended that children should not exceed in their daily dietary intake? Free sugars are any sugars added to food or drinks, or found naturally in honey, syrups and unsweetened fruit juices.

required HARD

1 ○ Yes
2 ○ No
3 ○ Don’t know
8 *Skipped*
9 *Not Asked*

# Page: child_freesugar if q9a==1

<div class="alert alert-error">Please only select one of these options</div>

<div class="alert alert-error">Please select one of these options</div>

Please type in how much added or free sugars it is recommended that a child should not exceed as part of their daily dietary energy intake.</br><br/>Please use just one of the boxes below .

**q9b** OPEN REAL TEXTBOX

Please type in the percentage (%).

| | |
|---|---|
| required | NONE |
| max | 100 |
| min | 0 |
| right | % |

<b> Or</b>

**q9c** OPEN INTEGER TEXTBOX

Please type in the number of grams of sugar per day.

| | |
|---|---|
| required | NONE |
| min | 0 |
| max | 10000 |
| right | g |

<b> Or</b>

**q9d** OPEN REAL TEXTBOX

Please type in the number of teaspoons of sugar per day.

| | |
|---|---|
| required | NONE |
| max | 1000 |
| min | 0 |
| right | tsp |

## Page: implicit_page_q10

**q10-*required*** SINGLE CHOICE

Which do you think has more energy per gram: sugar (e.g. caster sugar) or fat (e.g. in butter)?

| | |
|---|---|
| required | HARD |
| order | randomize |

1 ○ Sugar
2 ○ Fat
3 ○ Don’t know/ not sure *Not randomized*
8 *Skipped*
9 *Not Asked*

end module: implicit_module_1

# Module: sugar

# Page: cola

<img src="$teaspoon" ></br> Free sugars are any sugars added to food or drinks, or found naturally in honey, syrups and unsweetened fruit juices.</br></br>How many teaspoons of added or free sugars do you think are in ...</br> <img src="$cola"  >

**q11a-** ***required*** OPEN INTEGER TEXTBOX

Can of cola (330 ml)

| | |
|---|---|
| required | HARD |
| dk | 1 |
| cols | 5 |

# Page: ice

<img src="$teaspoon" ></br> Free sugars are any sugars added to food or drinks, or found naturally in honey, syrups and unsweetened fruit juices.</br></br>How many teaspoons of added or free sugars do you think are in ...</br> <img src="$ice_cream" >

**q11b-** ***required*** OPEN INTEGER TEXTBOX

Chocolate milkshake (330ml)

| | |
|---|---|
| required | HARD |
| dk | 1 |
| cols | 5 |

# Page: choco

<img src="$teaspoon" ></br> Free sugars are any sugars added to food or drinks, or found naturally in honey, syrups and unsweetened fruit juices.</br></br>How many teaspoons of added or free sugars do you think are in ...</br> <img src="$juice"  >

**q11c-** ***required*** OPEN INTEGER TEXTBOX

Orange juice (330ml)

| | |
|---|---|
| required | HARD |
| dk | 1 |
| cols | 5 |

# Page: latte

<img src="$teaspoon" ></br> Free sugars are any sugars added to food or drinks, or found naturally in honey, syrups and unsweetened fruit juices.</br></br>How many teaspoons of added or free sugars do you think are in ...</br> <img src="$latte" >

**q11d-*required***

OPEN INTEGER TEXTBOX

Latte Coffee with vanilla syrup (330ml)

| | |
|---|---|
| required | HARD |
| dk | 1 |
| cols | 5 |

## Page: ginger

<img src="$teaspoon" ></br> Free sugars are any sugars added to food or drinks, or found naturally in honey, syrups and unsweetened fruit juices.</br></br>How many teaspoons of added or free sugars do you think are in ...</br> <img src="$ginger" >

**q11e-*required***

OPEN INTEGER TEXTBOX

Can of ginger beer (330 ml)

| | |
|---|---|
| required | HARD |
| dk | 1 |
| cols | 5 |

## Page: energy

<img src="$teaspoon" ></br> Free sugars are any sugars added to food or drinks, or found naturally in honey, syrups and unsweetened fruit juices.</br></br>How many teaspoons of added or free sugars do you think are in ...</br> <img src="$energy" >

**q11f-*required***

OPEN INTEGER TEXTBOX

Can of energy drink (330ml)

| | |
|---|---|
| required | HARD |
| dk | 1 |
| cols | 5 |

end module: sugar

# Module: implicit_module_2

# Page: implicit_page_q12

**q12-**
***required***

OPEN TEXTBOX

Which, if any, health conditions do you think can result from being obese/ overweight?

| | |
|---|---|
| required | HARD |
| rows | 4 |
| cols | 60 |

# Page: implicit_page_q13

**q13-**
***required***

MULTIPLE CHOICE

Which, if any, of the following health conditions do you think can result from being overweight/ obese?

| | |
|---|---|
| required | HARD |
| order | randomize |

| | | | |
|---|---|---|---|
| 1 | ☐ | Cancer | |
| 2 | ☐ | Diabetes | |
| 3 | ☐ | Arthritis | |
| 4 | ☐ | Heart disease | |
| 5 | ☐ | Stroke | |
| 6 | ☐ | Flu | |
| 7 | ☐ | Shingles | |
| 98 | ☐ | None of these | *Not randomized,exclude other punches* |
| 99 | ☐ | Don't know / not sure | *Not randomized,exclude other punches* |

# Page: implicit_page_q14

**q14-** ***required*** GRID

Being overweight or obese can increase a person’s chance of developing certain types of cancer. For each of the following types of cancer, please say whether or not you think being overweight/obese could increase a person’s chance of developing it.

| | |
|---|---|
| required | HARD |
| displaymax | 5 |
| roworder | randomize |

ROWS

| | |
|---|---|
| q14_1- *required* | Bowel |
| q14_2- *required* | Kidney |
| q14_3- *required* | Oesophageal |
| q14_4- *required* | Pancreatic |
| q14_5- *required* | Gall bladder |
| q14_6- *required* | Advanced prostate |
| q14_7- *required* | Breast (postmenopausal) |
| q14_8- *required* | Womb |
| q14_9- *required* | Ovarian |
| q14_10- *required* | Stomach |
| q14_11- *required* | Liver |
| q14_12- *required* | Cervical |
| q14_13- *required* | Bladder |

COLUMNS

1 ○ Yes it could
2 ○ No it couldn't
99 ○ Don't know / not sure
8 *Skipped*
9 *Not Asked*

## Page: implicit_page_q16a

**q16a-** ***required*** DROPDOWN

What is your weight? If you are not sure please pick your best estimate. If you would prefer not to say then that option is at the bottom of the list.

required HARD

1 ○ Less than 6st
2 ○ 6st 0lb / 38.1kg
3 ○ 6st 1lb / 38.6kg
4 ○ 6st 2lb / 39.0kg
5 ○ 6st 3lb / 39.5kg
6 ○ 6st 4lb / 39.9kg
7 ○ 6st 5lb / 40.4kg
8 ○ 6st 6lb / 40.8kg
9 ○ 6st 7lb / 41.3kg
10 ○ 6st 8lb / 41.7kg
11 ○ 6st 9lb / 42.2kg
12 ○ 6st 10lb / 42.6kg
13 ○ 6st 11lb / 43.1kg
14 ○ 6st 12lb / 43.5kg
15 ○ 6st 13lb / 44.0kg
16 ○ 7st 0lb / 44.5kg
17 ○ 7st 1lb / 44.9kg
18 ○ 7st 2lb / 45.4kg
19 ○ 7st 3lb / 45.8kg
20 ○ 7st 4lb / 46.3kg
21 ○ 7st 5lb / 46.7kg
22 ○ 7st 6lb / 47.2kg
23 ○ 7st 7lb / 47.6kg
24 ○ 7st 8lb / 48.1kg
25 ○ 7st 9lb / 48.5kg
26 ○ 7st 10lb / 49kg
27 ○ 7st 11lb / 49.4kg
28 ○ 7st 12lb / 49.9kg
29 ○ 7st 13lb / 50.3kg
30 ○ 8st 1lb / 51.3kg
31 ○ 8st 2lb / 51.7kg
32 ○ 8st 3lb / 52.2kg
33 ○ 8st 4lb / 52.6kg
34 ○ 8st 5lb / 53.1kg
35 ○ 8st 6lb / 53.5kg
36 ○ 8st 7lb / 54kg
37 ○ 8st 8lb / 54.4kg
38 ○ 8st 9lb / 54.9kg
39 ○ 8st 10lb / 55.3kg
40 ○ 8st 11lb / 55.8kg
41 ○ 8st 12lb / 56.2kg
42 ○ 8st 13lb / 56.7kg
43 ○ 9st 0lb / 57.2kg
44 ○ 9st 1lb / 57.6kg
45 ○ 9st 2lb / 58.1kg
46 ○ 9st 3lb / 58.5kg
47 ○ 9st 4lb / 59kg
48 ○ 9st 5lb / 59.4kg

49 ○ 9st 6lb / 59.9kg
50 ○ 9st 7lb / 60.3kg
51 ○ 9st 8lb / 60.8kg
52 ○ 9st 9lb / 61.2kg
53 ○ 9st 10lb / 61.7kg
54 ○ 9st 11lb / 62.1kg
55 ○ 9st 12lb / 62.6kg
56 ○ 9st 13lb / 63kg
57 ○ 10st 0lb / 63.5kg
58 ○ 10st 1lb / 64kg
59 ○ 10st 2lb / 64.4kg
60 ○ 10st 3lb / 64.9kg
61 ○ 10st 4lb / 65.3kg
62 ○ 10st 5lb / 65.8kg
63 ○ 10st 6lb / 66.2kg
64 ○ 10st 7lb / 66.7kg
65 ○ 10st 8lb / 67.1kg
66 ○ 10st 9lb / 67.6kg
67 ○ 10st 10lb / 68kg
68 ○ 10st 11lb / 68.5kg
69 ○ 10st 12lb / 68.9kg
70 ○ 10st 13lb / 69.4kg
71 ○ 11st 0lb / 69.9kg
72 ○ 11st 1lb / 70.3kg
73 ○ 11st 2lb / 70.8kg
74 ○ 11st 3lb / 71.2kg
75 ○ 11st 4lb / 71.7kg
76 ○ 11st 5lb / 72.1kg
77 ○ 11st 6lb / 72.6kg
78 ○ 11st 7lb / 73kg
79 ○ 11st 8lb / 73.5kg
80 ○ 11st 9lb / 73.9kg
81 ○ 11st 10lb / 74.4kg
82 ○ 11st 11lb / 74.8kg
83 ○ 11st 12lb / 75.3kg
84 ○ 11st 13lb / 75.7kg
85 ○ 12st 0lb / 76.2kg
86 ○ 12st 1lb / 76.7kg
87 ○ 12st 2lb / 77.1kg
88 ○ 12st 3lb / 77.6kg
89 ○ 12st 4lb / 78kg
90 ○ 12st 5lb / 78.5kg
91 ○ 12st 6lb / 78.9kg
92 ○ 12st 7lb / 79.4kg
93 ○ 12st 8lb / 79.8kg
94 ○ 12st 9lb / 80.3kg
95 ○ 12st 10lb / 80.7kg
96 ○ 12st 11lb / 81.2kg

97 ○ 12st 12lb / 81.6kg
98 ○ 12st 13lb / 82.1kg
99 ○ 13st 0lb / 82.6kg
100 ○ 13st 1lb / 83kg
101 ○ 13st 2lb / 83.5kg
102 ○ 13st 3lb / 83.9kg
103 ○ 13st 4lb / 84.4kg
104 ○ 13st 5lb / 84.8kg
105 ○ 13st 6lb / 85.3kg
106 ○ 13st 7lb / 85.7kg
107 ○ 13st 8lb / 86.2kg
108 ○ 13st 9lb / 86.6kg
109 ○ 13st 10lb / 87.1kg
110 ○ 13st 11lb / 87.5kg
111 ○ 13st 12lb / 88kg
112 ○ 13st 13lb / 88.5kg
113 ○ 14st 0lb / 88.9kg
114 ○ 14st 1lb / 89.4kg
115 ○ 14st 2lb / 89.8kg
116 ○ 14st 3lb / 90.3kg
117 ○ 14st 4lb / 90.7kg
118 ○ 14st 5lb / 91.2kg
119 ○ 14st 6lb / 91.6kg
120 ○ 14st 7lb / 92.1kg
121 ○ 14st 8lb / 92.5kg
122 ○ 14st 9lb / 93kg
123 ○ 14st 10lb / 93.4kg
124 ○ 14st 11lb / 93.9kg
125 ○ 14st 12lb / 94.3kg
126 ○ 14st 13lb / 94.8kg
127 ○ 15st 0lb / 95.3kg
128 ○ 15st 1lb / 95.7kg
129 ○ 15st 2lb / 96.2kg
130 ○ 15st 3lb / 96.6kg
131 ○ 15st 4lb / 97.1kg
132 ○ 15st 5lb / 97.5kg
133 ○ 15st 6lb / 98kg
134 ○ 15st 7lb / 98.4kg
135 ○ 15st 8lb / 98.9kg
136 ○ 15st 9lb / 99.3kg
137 ○ 15st 10lb / 99.8kg
138 ○ 15st 11lb / 100.2kg
139 ○ 15st 12lb / 100.7kg
140 ○ 15st 13lb / 101.1kg
141 ○ 16st 1lb / 102.1kg
142 ○ 16st 2lb / 102.5kg
143 ○ 16st 3lb / 103kg
144 ○ 16st 4lb / 103.4kg

145 ○ 16st 5lb / 103.9kg
146 ○ 16st 6lb / 104.3kg
147 ○ 16st 7lb / 104.8kg
148 ○ 16st 8lb / 105.2kg
149 ○ 16st 9lb / 105.7kg
150 ○ 16st 10lb / 106.1kg
151 ○ 16st 11lb / 106.6kg
152 ○ 16st 12lb / 107kg
153 ○ 16st 13lb / 107.5kg
154 ○ 17st 0lb / 108kg
155 ○ 17st 1lb / 108.4kg
156 ○ 17st 2lb / 108.9kg
157 ○ 17st 3lb / 109.3kg
158 ○ 17st 4lb / 109.8kg
159 ○ 17st 5lb / 110.2kg
160 ○ 17st 6lb / 110.7kg
161 ○ 17st 7lb / 111.1kg
162 ○ 17st 8lb / 111.6kg
163 ○ 17st 9lb / 112kg
164 ○ 17st 10lb / 112.5kg
165 ○ 17st 11lb / 112.9kg
166 ○ 17st 12lb / 113.4kg
167 ○ 17st 13lb / 113.9kg
168 ○ 18st 0lb / 114.3kg
169 ○ 18st 1lb / 114.8kg
170 ○ 18st 2lb / 115.2kg
171 ○ 18st 3lb / 115.7kg
172 ○ 18st 4lb / 116.1kg
173 ○ 18st 5lb / 116.6kg
174 ○ 18st 6lb / 117kg
175 ○ 18st 7lb / 117.5kg
176 ○ 18st 8lb / 117.9kg
177 ○ 18st 9lb / 118.4kg
178 ○ 18st 10lb / 118.8kg
179 ○ 18st 11lb / 119.3kg
180 ○ 18st 12lb / 119.7kg
181 ○ 18st 13lb / 120.2kg
182 ○ 19st 0lb / 120.7kg
183 ○ 19st 1lb / 121.1kg
184 ○ 19st 2lb / 121.6kg
185 ○ 19st 3lb / 122kg
186 ○ 19st 4lb / 122.5kg
187 ○ 19st 5lb / 122.9kg
188 ○ 19st 6lb / 123.4kg
189 ○ 19st 7lb / 123.8kg
190 ○ 19st 8lb / 124.3kg
191 ○ 19st 9lb / 124.7kg
192 ○ 19st 10lb / 125.2kg

193 ○ 19st 11lb / 125.6kg
194 ○ 19st 12lb / 126.1kg
195 ○ 19st 13lb / 126.6kg
196 ○ More than 20st
197 ○ Don't know
198 ○ Prefer not to say
999 *Skipped*
999 *Not Asked*

## Page: implicit_page_height_metres

**height_metres-** ***Show if not pdl.height_metres/required*** DROPDOWN

How tall are you?
Please try to be as accurate as possible.
If you would prefer not to say - just select this option

| | |
|---|---|
| required | HARD |
| tags | height, BMI, metres |

99 ○ Prefer not to say
1 ○ 4 ft 0 in / 122 cm
2 ○ 4 ft 1 in / 124 cm
3 ○ 4 ft 2 in / 127 cm
4 ○ 4 ft 3 in / 130 cm
5 ○ 4 ft 4 in / 132 cm
6 ○ 4 ft 5 in / 135 cm
7 ○ 4 ft 6 in / 137 cm
8 ○ 4 ft 7 in / 140 cm
9 ○ 4 ft 8 in / 142 cm
10 ○ 4 ft 9 in / 145 cm
11 ○ 4 ft 10 in / 147 cm
12 ○ 4 ft 11 in / 150 cm
13 ○ 5 ft 0 in / 152 cm
14 ○ 5 ft 1 in / 155 cm
15 ○ 5 ft 2 in / 157 cm
16 ○ 5 ft 3 in / 160 cm
17 ○ 5 ft 4 in / 163 cm
18 ○ 5 ft 5 in / 165 cm
19 ○ 5 ft 6 in / 168 cm
20 ○ 5 ft 7 in / 170 cm
21 ○ 5 ft 8 in / 173 cm
22 ○ 5 ft 9 in / 175 cm
23 ○ 5 ft 10 in / 178 cm
24 ○ 5 ft 11 in / 180 cm
25 ○ 6 ft 0 in / 183 cm
26 ○ 6 ft 1 in / 185 cm
27 ○ 6 ft 2 in / 188 cm
28 ○ 6 ft 3 in / 191 cm
29 ○ 6 ft 4 in / 193 cm
30 ○ 6 ft 5 in / 196 cm
31 ○ 6 ft 6 in / 198 cm
32 ○ 6 ft 7 in / 201 cm
33 ○ 6 ft 8 in / 203 cm
34 ○ 6 ft 9 in / 206 cm
35 ○ 6 ft 10 in / 208 cm
36 ○ 6 ft 11 in / 211 cm
37 ○ 7 ft 0 in / 213 cm
38 ○ 7 ft 1 in / 216 cm
39 ○ 7 ft 2 in / 218 cm
40 ○ 7 ft 3 in / 221 cm
41 ○ 7 ft 4 in / 224 cm
42 ○ 7 ft 5 in / 226 cm
43 ○ 7 ft 6 in / 229 cm
44 ○ 7 ft 7 in / 231 cm
45 ○ 7 ft 8 in / 234 cm
46 ○ 7 th 9 in / 236 cm
47 ○ 7 th 10 in / 239 cm

48 ○ 7 ft 11 in / 241 cm
49 ○ Don't know
998 *Skipped*
999 *Not Asked*

# Page: implicit_page_q18

**q18-*required*** SINGLE CHOICE

Considering your current weight do you believe you are...?

required HARD

1 ○ Very underweight
2 ○ Slightly underweight
3 ○ About the right weight
4 ○ Slightly overweight
5 ○ Very overweight
99 ○ Don't know
998 *Skipped*
999 *Not Asked*

# Page: implicit_page_q19

**q19-*required*** SINGLE CHOICE

In the past 12 months, has a doctor, nurse, or other healthcare professional given you advice about your weight?

required HARD

1 ○ No
2 ○ Yes, lose weight
3 ○ Yes, gain weight
4 ○ Yes, maintain current weight
96 ○ Other advice (open [q19_open])
98 ○ Not applicable, have not seen a healthcare professional in the past 12 months *Exclude other punches*
99 ○ Don't know/ can't remember *Exclude other punches*
998 *Skipped*
999 *Not Asked*

# Page: implicit_page_q20

***q20-required*** SINGLE CHOICE

The following question relates to tobacco smoking (e.g. cigarettes, pipes, cigars or other tobacco products), but not electronic cigarettes or other nicotine replacement products (because these do not contain tobacco)

Which of the following best describes your smoking status?

required HARD

1 ○ I have never smoked
2 ○ I used to smoke but I have given up now
3 ○ I smoke but I don't smoke every day
4 ○ I smoke every day
8 *Skipped*
9 *Not Asked*

# Page: implicit_page_q21

***q21-required*** SINGLE CHOICE

How often do you have a drink containing alcohol?

required HARD

1 ○ Never
2 ○ Monthly or less
3 ○ 2 to 4 times a month
4 ○ 2 to 3 times a week
5 ○ 4 or more times a week
8 *Skipped*
9 *Not Asked*

# Page: alcohol if q21!=1

<img src="$units" >

**q22-** ***required*** SINGLE CHOICE

How many units of alcohol do you drink on a typical day when you are drinking?

required HARD

1 ○ 1 or 2
2 ○ 3 or 4
3 ○ 5 or 6
4 ○ 7, 8, or 9
5 ○ 10 or more
8 *Skipped*
9 *Not Asked*

# Page: implicit_page_q23

**q23-** ***required*** GRID

How many people of these age groups live in your household on a regular basis?

required HARD

ROWS

| | |
|---|---|
| q23_1- *required* | Number of adults, aged 18 or older, including yourself |
| q23_2- *required* | Number of teenagers aged between 13 and 17 |
| q23_3- *required* | Number of children aged 12 or less |

COLUMNS

1 ○ 0
2 ○ 1
3 ○ 2
4 ○ 3
5 ○ 4 or more
8 *Skipped*
9 *Not Asked*

# Page: end

<b><ul>Thank you for completing the survey. If you'd like to find out more about the issues of weight, smoking and drinking in this survey there are a number of websites with advice:<br/><br/><li>Causes of cancer and reducing your risk - www.cancerresearchuk.org/about-cancer/causes-of-cancer</li><li>Quit smoking - www.nhs.uk/smokefree</li><li>NHS - Live Well - www.nhs.uk/livewell/pages/livewellhub.aspx</li><li> Drinkline on 0300 123 1110 (Mon-Fri 9 am - 8 pm, weekends 11 am - 4 pm) for concern about alcohol.</li></ul><br/> Please click the arrow below to proceed</b>

end module: implicit_module_2
